# Gene expression profiles analysis identifies a novel two-gene signature to predict overall survival in diffuse large B-cell lymphoma

**DOI:** 10.1042/BSR20181293

**Published:** 2019-01-03

**Authors:** Chengtao Sun, Xianfeng Cheng, Chaoyu Wang, Xi Wang, Bing Xia, Yizhuo Zhang

**Affiliations:** 1 Department of Hematology, Tianjin Medical University Cancer Institute and Hospital; National Clinical Research Center for Cancer; Key Laboratory of Cancer Prevention and Therapy, Tianjin; Tianjin’s Clinical Research Center for Cancer, Huanhuxi Road, Tianjin 300060, P.R. China; 2 Department of Cardiovascular Surgery, Weifang People’s Hospital, Weifang 261041, Shandong, P.R. China

**Keywords:** bioinformatics analysis, Diffuse large B cell lymphoma, differentially expressed genes, protein-protein interaction network, survival analysis

## Abstract

Diffuse large B-cell lymphoma (DLBCL) is the most common hematologic malignancy, however, specific tumor-associated genes and signaling pathways are yet to be deciphered. Differentially expressed genes (DEGs) were computed based on gene expression profiles from GSE32018, GSE56315, and The Cancer Genome Atlas (TCGA) DLBC. Overlapping DEGs were then evaluated for gene ontology (GO), pathways enrichment, DNA methylation, protein–protein interaction (PPI) network analysis as well as survival analysis. Seventy-four up-regulated and 79 down-regulated DEGs were identified. From PPI network analysis, majority of the DEGs were involved in cell cycle, oocyte meiosis, and epithelial-to-mesenchymal transition (EMT) pathways. Six hub genes including *CDC20, MELK, PBK*, prostaglandin D2 synthase (*PTGDS*), *PCNA*, and *CDK1* were selected using the Molecular Complex Detection (MCODE). *CDC20* and *PTGDS* were able to predict overall survival (OS) in TCGA DLBC and in an additional independent cohort GSE31312. Furthermore, *CDC20* DNA methylation negatively regulated *CDC20* expression and was able to predict OS in DLBCL. A two-gene panel consisting of *CDC20* and *PTGDS* had a better prognostic value compared with *CDC20* or *PTGDS* alone in the TCGA cohort (*P*=0.026 and 0.039). Overall, the present study identified a set of novel genes and pathways that may play a significant role in the initiation and progression of DLBCL. In addition, *CDC20* and *PTGDS* will provide useful guidance for therapeutic applications.

## Introduction

Diffuse large B-cell lymphoma (DLBCL), the most frequently diagnosed subtype of hematological cancers, is a molecular heterogeneous disease with an annual incidence rate of over 100000 cases worldwide [1,2]. Although DLBCL is a curable lymphoma [3,4], up to 40% of patients succumb to this cancer, thus indicating the pathology and mechanism of DLBCL remains ambiguous. Deciphering the genes and signaling pathways modulated during tumorigenesis will help guide therapeutic efficacy.

High-throughput sequencing including gene-chip and next-generation sequencing (NGS) is a rapid and efficient method to obtain differentially expressed genes (DEGs) between tumor and normal tissues [3,4]. At present, the high-throughput data of various genomic alterations in various cancers have been generated and archived in public databases. Recent studies have identified hundreds of DEGs in DLBCL [5]. However, the results of these studies have contradictory or inconsistent data due to small sample size [6], tissue heterogeneity or produced from single cohort studies [7]. Integrating and re-analyzing published sequence data may help resolve these issues. Gene Expression Omnibus (GEO) and The Cancer Genome Atlas (TCGA) are two available public databases that provide the opportunity to investigate new research based on gene sequencing data mining with large-scale clinical samples from multiple cohorts.

In the current study, we initially identified 153 overlapping DEGs from GSE32018 [8], GSE56315 [9] and TCGA DLBC datasets. Gene ontology (GO) and pathways enrichment, as well as protein–protein interaction (PPI) network of these DEGs were performed. Based on Molecular Complex Detection (MCODE) algorithm, hub genes were selected and their prognostic role was evaluated based on TCGA DLBC. In addition, we successfully validated the prognostic signature of these hub genes using an independent cohort from GSE31312 [10]. We also constructed a two-gene combined panel and confirmed this model as a more sensitive predictive tool.

## Materials and methods

### Gene expression profile datasets and DEGs identification

The DLBCL and noncancerous tissues gene expression profile datasets GSE32018 and GSE56315 were obtained from NCBI-GEO (https://www.ncbi.nlm.nih.gov/geo/). GEO datasets platforms were GPL570 (Affymetrix Human Genome U133 Plus 2.0 Array, Affymetrix, Santa Clara, CA, U.S.A.) for GSE56315 and GPL6480 and (Agilent-014850 Whole Human Genome Microarray 4x44K G4112F, Agilent Technologies, Santa Clara, CA, U.S.A.) for GSE32018. GSE56315 dataset included 89 DLBCL tissues and 33 normal tonsil tissues. As for the GSE32018 dataset, 22 DLBCL tissues and 7 normal lymph nodes were profiled. High-throughput data of RNA-Seq for patients diagnosed with DLBCL were downloaded from TCGA (https://tcga-data.nci.nih.gov/tcga/). RNA-Seq data from Illumina HiSeq RNASeq platform comprised 48 DLBCL tissues.

The R language package DESeq was used for determining DEGs between DLBCL samples and noncancerous tissues (adjusted *P*<0.01 and [logFC] > 1 as the cut-off criterion), respectively. The FunRich [11] software was used for analysis of DEGs that overlapped between the two GEO datasets.

### GO and pathway enrichment analysis

Candidate DEGs function and pathway enrichment were analyzed using the Database for Annotation, Visualization and Integrated Discovery (DAVID, version 6.7, https://david.ncifcrf.gov/) and FUNRICH Software. *P*<0.05 was defined as the cutoff for significant function and pathway analysis.

### Integration of PPI network, hub genes, and significant pathway identification

The Search Tool for the Retrieval of Interacting Genes (STRING) database (version 10.0, http://string-db.org) was used to predict candidate DEG-encoded PPI. Afterward, Cytoscape software (version 3.4.0, http://www.cytoscape.org/) was used to construct the PPI network. In addition, MCODE was used to analyze PPI network modules [12]. DAVID and FUNRICH were used to perform pathway enrichment analysis of gene modules. At last, hub genes were identified using the MCODE plug-in, and was used to calculate node degree, i.e. the number of interconnections to filter hub genes of PPI.

### Validation of the diagnostic effectiveness of the identified hub genes for DLBCL

The receiver operating characteristic (ROC) curve was used to assess the diagnostic effectiveness of the identified hub genes between DLBCL and normal tissue, and was based on the GSE56315 dataset.

### Bioinformatics analysis of the association between hub gene expression and overall survival in patients with DLBCL

The association between the identified hub gene expression and overall survival (OS) for DLBCL patients was assessed using data from TCGA DLBC and GSE3131. Data for hub gene expression and OS from TCGA DLBC were downloaded from the UCSC Xena browser (https://xenabrowser.net). Forty patients with complete clinical stage and OS data were selected and analyzed using SPSS 20. (SPSS Inc., Chicago, IL, U.S.A.) and *P*-values <0.05 were considered statistically significant. The R2 web-based application (http://r2.amc.nl) was used to generate Kaplan–Meier survival curves from data in GSE31312. The optimal cutoff was selected by scan model. Kaplan–Meier curves for OS were generated using the auto-select best cutoff.

In addition, the prognostic potential of the two-gene panel was determined by combining *CDC20* and prostaglandin D2 synthase (*PTGDS*) and compared with *CDC20* or *PTGDS* alone using ROC analysis. All statistical analyses were performed using SPSS 20 (SPSS Inc., Chicago, IL, U.S.A.). *P*-values <0.05 were considered statistically significant.

### Bioinformatics analysis of the association between *CDC20* and *PTGDS* methylation and OS in DLBCL patients

RNA-seq and Illumina 450 K methylation array datasets were downloaded using the UCSC Xena browser for TCGA DLBC. The *CDC20* and *PTGDS* mRNA expression and their DNA methylation levels were obtained by data mining the TCGA DLBC. The association between *CDC20* and *PTGDS* mRNA expression, *CDC20* and *PTGDS* methylation status and OS in DLBCL patients were analyzed using SPSS 20 (SPSS Inc., Chicago, IL, U.S.A.) and *P*-values <0.05 were considered statistically significant.

## Results

### Identification of overlapping DEGs in DLBCL

**Figure 1:**
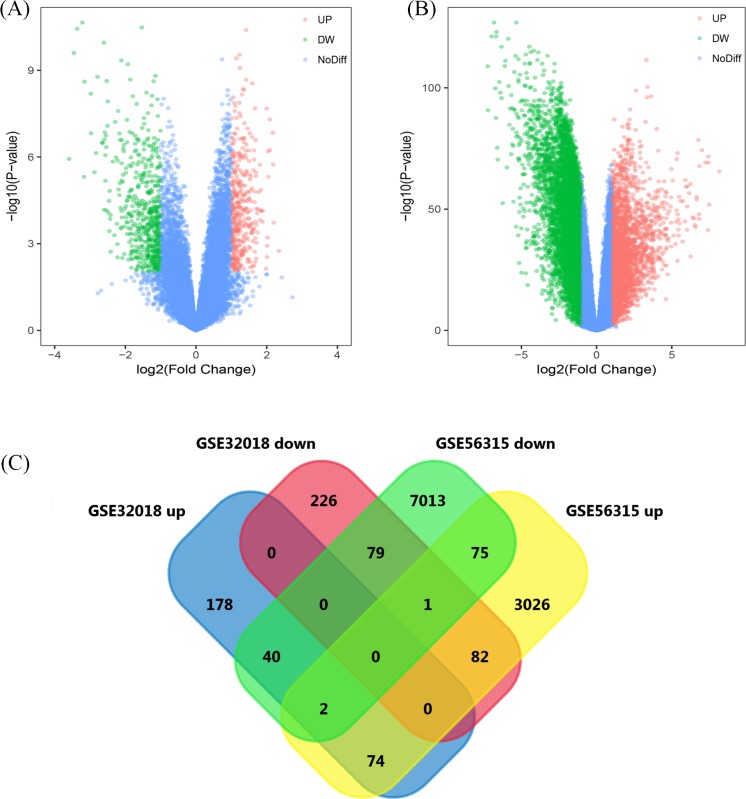
Volcano plots and Venn diagram for microarray data on DEGs between DLBCL and noncancerous tissues (**A**) Based on GSE32018 and (**B**) GSE56315, red dots indicate up-regulated and green dots indicate down-regulated DEGs. Blue dots denote genes that are not differentially expressed. The x-axis represents the value of log_2_FC and the y-axis represents transformed (−log10) FDR. (**C**) Venn diagram of DEGs for GSE32018 genes that are up-regulated and down-regulated; GSE56315 up-regulated and down-regulated genes. The cross areas denote overlapping DEGs.

Based on the cut-off criteria of *P*<0.01 and [logFC] > 1 for selecting DEGs, a total of 295 and 3017 up-regulated DEGs, 387 and 6613 down-regulated DEGs were identified from GSE32018 and GSE56315, respectively (Figure 1A,B). As shown in Figure 1C, 74 up-regulated genes and 79 down-regulated genes overlapped between the two datasets.

### Functional enrichment analysis of DEGs in DLBCL

**Figure 2:**
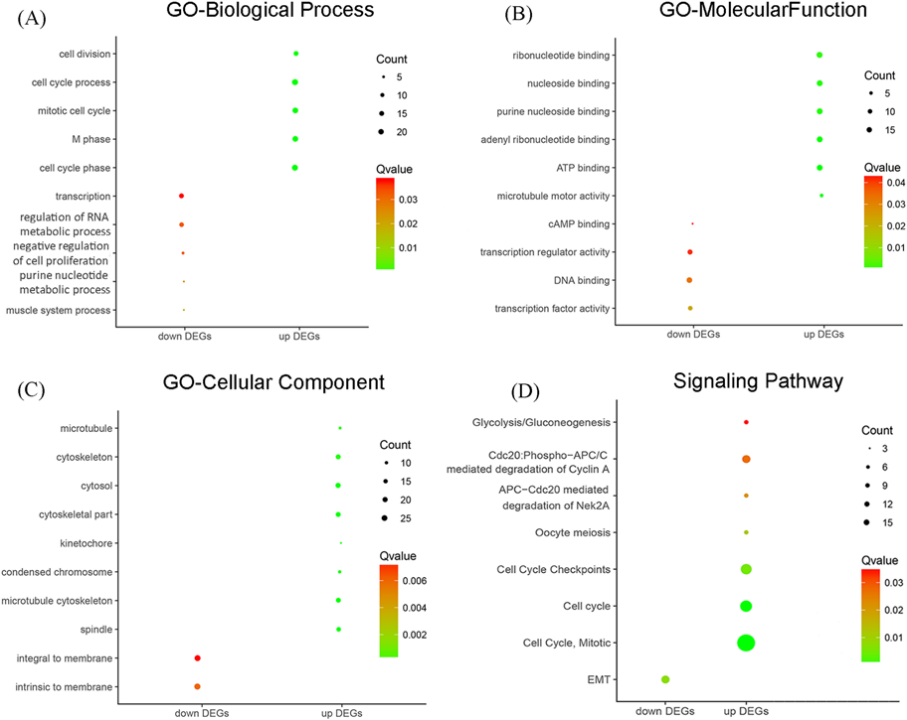
Significantly enriched GO and pathway terms of the DEGs in DLBCL (**A**–**C**) were significantly enriched for BP, MF, and CC for the up-regulated and down-regulated DEGs in DLBCL. (**D**) Significant enriched pathways of the up-regulated and down-regulated DEGs in DLBCL.

As shown in Figure 2A and Table 1, GO enriched functions for the 74 overlapped up-regulated genes and 79 overlapped down-regulated genes were involved in a number of biological processes (BP), including cell cycle phase, M phase, mitotic cell cycle, cell cycle process, and cell division for the up-regulated genes, and muscle system process, purine nucleotide metabolic process, negative regulation of cell proliferation, regulation of RNA metabolic process, and transcription for the down-regulated genes. With regard to molecular function (MF), the top six MFs of the up-regulated DEGs were microtubule motor activity, ATP binding, adenyl ribonucleotide binding, purine nucleoside binding, nucleoside binding, and ribonucleotide binding, and the top four MFs of the down-regulated DEGs were transcription factor activity, DNA binding, transcription regulator activity, and cAMP binding (Figure 2B and Table 1). For the cellular component (CC) terms, majority of the up-regulated DEGs were enriched for spindle, microtubule cytoskeleton, condensed chromosome, kinetochore, cytoskeletal part, cytosol, cytoskeleton, and microtubule. For the majority of the down-regulated genes were enriched for ‘intrinsic to membrane’ and ‘integral to membrane’ (Figure 2C and Table 1). Furthermore, the up-regulated genes were largely enriched for cell cycle, glycolysis/gluconeogenesis, oocyte meiosis, cell cycle mitotic, cell cycle checkpoints, APC-Cdc20-mediated degradation of Nek2A and cdc20: p-APC/C-mediated degradation of cyclin A pathways. The down-regulated genes were significantly enriched for epithelial-to-mesenchymal transition (EMT) pathway (Figure 2D and Table 2).

**Table 1:** GO analysis of DEGs associated with DLBCL

Term	Description	Count	*P*-value
Up-regulated			
GO:0022403	Cell cycle phase	21	2.69E-15
GO:0000279	M phase	19	9.84E-15
GO:0000278	Mitotic cell cycle	19	7.50E-14
GO:0022402	Cell cycle process	22	8.22E-14
GO:0003777	Microtubule motor activity	5	3.33E-04
GO:0005524	ATP binding	17	3.58E-04
GO:0032559	Adenyl ribonucleotide binding	17	4.17E-04
GO:0001883	Purine nucleoside binding	17	8.80E-04
GO:0001882	Nucleoside binding	17	9.49E-04
GO:0005819	Spindle	14	2.04E-14
GO:0015630	Microtubule cytoskeleton	16	3.56E-09
GO:0000793	Condensed chromosome	9	5.24E-08
GO:0044430	Cytoskeletal part	17	8.49E-07
GO:0000776	Kinetochore	7	7.19E-07
GO:0005874	Microtubule	8	1.28E-04
Down-regulated			
GO:0006163	Purine nucleotide metabolic process	4	0.023786
GO:0008285	Negative regulation of cell proliferation	5	0.031481
GO:0051252	Regulation of RNA metabolic process	12	0.032176
GO:0006350	Transcription	13	0.038040
GO:0003700	Transcription factor activity	9	0.022489
GO:0003677	DNA binding	15	0.032567
GO:0030528	Transcription regulator activity	11	0.041292

**Table 2:** Signaling pathway enrichment analysis of DEGs associated with DLBCL

Pathway	Name	Gene count	*P*-value	Genes
Up-regulated DEG				
REACTOME: REACT_152	Cell cycle, mitotic	16	5.20E-08	*GINS1, KIF23, CDK1, SGOL2, DBF4, NUF2, CDC20, MCM10, KIF2C, CDCA8, MAD2L1, ZWINT, RRM2, PCNA, BUB1B, SKA1*
KEGG: hsa04110	Cell cycle	7	2.36E-05	*CDK1, MAD2L1, DBF4, PCNA, BUB1B, TTK, CDC20*
REACTOME: REACT_1538	Cell cycle checkpoints	6	0.004610	*CDK1, MAD2L1, DBF4, BUB1B, CDC20, MCM10*
KEGG: hsa04114	Oocyte meiosis	3	0.010084	*CDK1, MAD2L1, CDC20*
REACTOME: REACT_8017	APC-Cdc20-mediated degradation of Nek2A	3	0.021560	*MAD2L1, BUB1B, CDC20*
REACTOME: REACT_6850	Cdc20:p-APC/C-mediated degradation of Cyclin A	4	0.027276	*CDK1, MAD2L1, BUB1B, CDC20*
KEGG: hsa00010	Glycolysis/gluconeogenesis	3	0.034518	*LDHA, PGAM1, GAPDH*
Down-regulated DEG				
FUNRICH biological pathway	EMT	4	0.007158	*PTGDS, FCER2, TPO*, IL11RA

### Hub genes and pathway identification with PPI network and modular analysis

**Figure 3:**
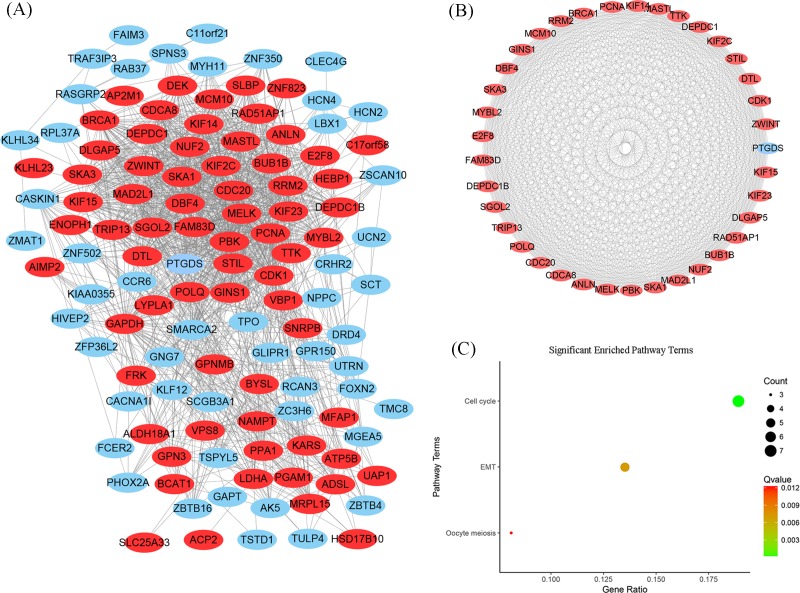
DEGs PPI network complex and modular analysis (**A**) Based on STRING and Cytoscape analysis, 69 up-regulated and 51 down-regulated DEGs were filtered into the PPI network complex. The black circle areas were the most significant modules. (**B**) The most significant module was identified using the Cytoscape MCODE plug-in, which consists of 37 nodes and 651 edges. (**C**) Significantly enriched pathway terms of the DEGs in the most significant module was associated with cell cycle, oocyte meiosis, and EMT. The size and color of the circles represent gene counts and Q values, respectively.

Using the STRING online database and Cytoscape software, a total of 120 DEGs (69 up-regulated and 51 down-regulated DEGs) of the 153 commonly altered DEGs were filtered into the DEGs PPI network, and contained 120 nodes and 1226 edges (Figure 3A). Thirty-three of the 153 DEGs did not fall into the DEGs PPI network. The entire PPI network was analyzed using MCODE, afterward, the most significant module was selected (Figure 3B). Pathway enrichment analysis showed that the most significant module consisted of 37 nodes and 651 edges (Figure 3B), and were mainly associated with cell cycle, oocyte meiosis, and EMT (Figure 3C and Table 3). The six most significant nodes from MCODE were *CDC20, MELK, PBK, PTGDS, PCNA*, and *CDK1*, and were identified as hub genes. All hub genes were up-regulated, except *PTGDS*.

**Table 3:** Pathway enrichment analysis of the most significant gene function modules

Term	Description	Count	*P*-value
KEGG cfa04110	Cell cycle	7	5.36E-09
FUNRICH biological pathway	EMT	5	0.007158
KEGG cfa04114	Oocyte meiosis	3	0.012116

### Validation of the diagnostic effectiveness of the six hub genes using GSE56315

**Figure 4:**
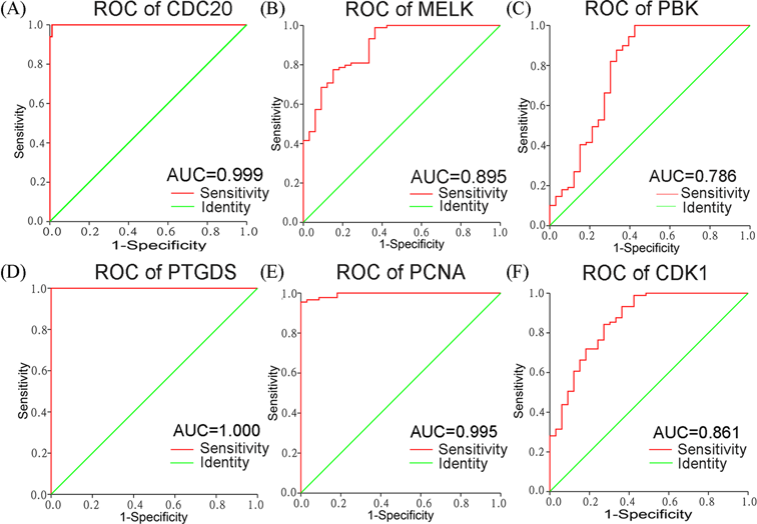
Validation of ROC results of the six hub genes in DLBCL based on GSE56315 (**A-F**) ROC curves of *CDC20, MELK, PBK, PTGDS, PCNA* and *CDK1* in GSE56315. Red represents sensitive curves, green indicates identify lines. The x-axis shows the false positive rate, and is presented as ‘1-Specificity’. The y-axis indicates true positive rate, and is shown as ‘Sensitivity’.

ROC analysis was performed from the six aberrantly expressed hub genes from GSE56315. The ROC curves of these six hub genes all indicated favorable diagnostic values for BLBCL (Figure 4 and Table 4). In addition, the area under ROC curve (AUC) of *PTGDS* and *CDC20* were 1.000 (*P*<0.01, Figure 4A and Table 4) and 0.999 (*P*<0.01, Figure 4D and Table 4), respectively.

**Table 4:** AUC of the six hub genes based on the GSE56315 dataset

Gene	AUC	95% CI	*P-*value
*CDC20*	0.999	0.997–1.000	<0.01
*MELK*	0.895	0.832–0.958	<0.001
*PBK*	0.786	0.676–0.896	<0.001
*PTGDS*	1.000	1.000–1.000	<0.01
*PCNA*	0.995	0.987–1.000	<0.01
*CDK1*	0.861	0.784–0.939	<0.01

Abbreviation: CI, confidence interval.

### High *CDC20* and low *PTGDS* expression may be an indicator for poor OS and a combined panel of these two genes is a superior sensitive predictive tool

**Figure 5:**
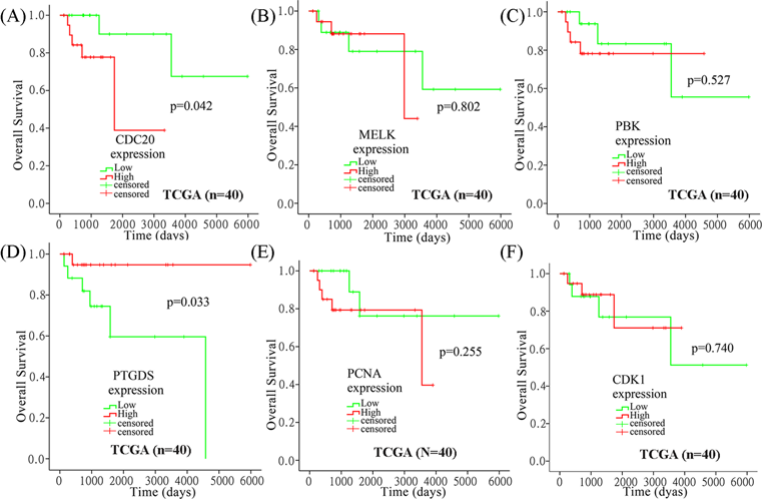
Kaplan–Meier curves of OS for patients with high or low hub gene expression in TCGA DLBC (**A**–**F**) Kaplan–Meier curves of OS for DLBCL patients with high or low *CDC20, MELK, PBK, PTGDS, PCNA*, and *CDK1* expression in TCGA DLBC. Analysis was performed using SPSS 20.0.

**Figure 6:**
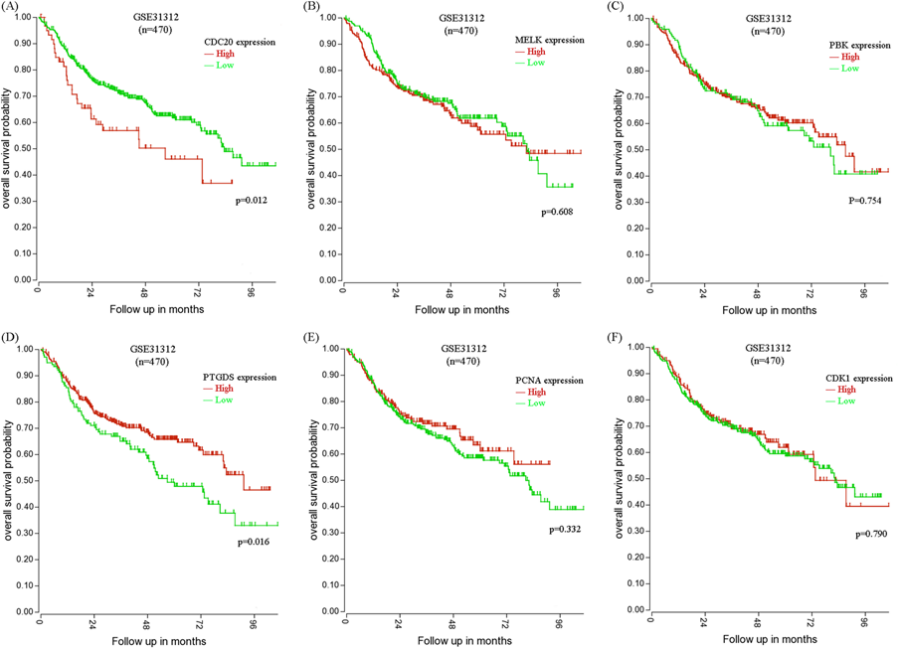
Kaplan–Meier curves of OS for DLBCL patients based on expression of the six hub genes in GSE31312 (**A**–**F**) Comparative OS between *CDC20, MELK, PBK, PTGDS, PCNA*, and *CDK1* higher and lower expression levels in GSE31312. Analysis was performed using R2.

By mining data from TCGA DLBC, we estimated the association between the expression of these six hub genes and OS in DLBCL patients using SPSS 20.0 (Figure 5). Our analysis demonstrated that high *CDC20* expression and low *PTGDS* expression were significantly associated with poor OS (*P*=0.042 and 0.033, respectively, Figure 5A,D), while the other four hub genes were not associated with OS (Figure 5B,C,E,F). To confirm our findings, data from GSE31312 (*n*=470) was analyzed using R2. Kaplan–Meier survival curves showed that *CDC20* and *PTGDS* had the same prognosis values as that observed for TCGA DLBC (*P*=0.012 and 0.016, respectively, Figure 6).

**Figure 7:**
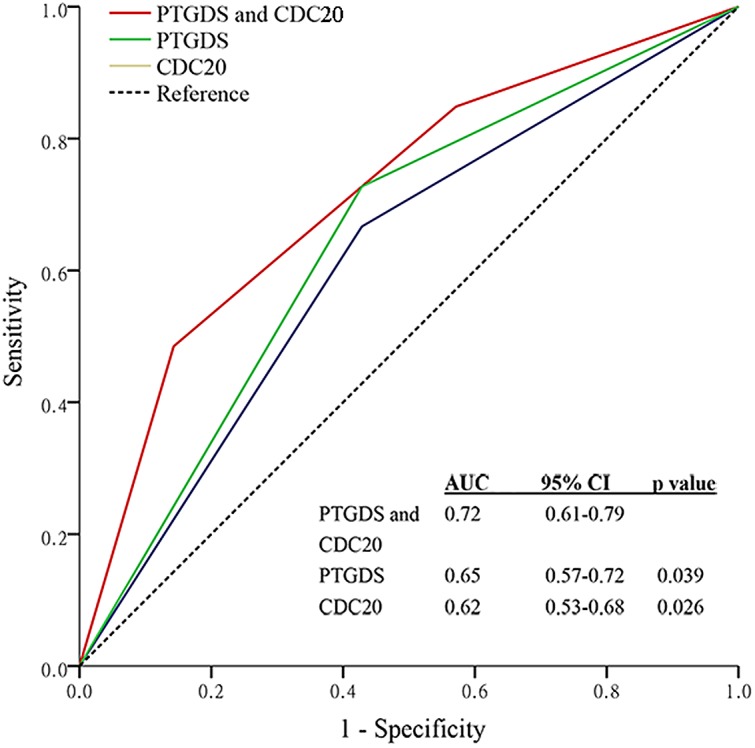
Comparisons of the sensitivity and specificity for survival prediction using the combined *CDC20* and *PTGDS* model, the *CDC20* alone model, or the *PTGDS* alone model Figure shows the ROC curves in the DLBCL cohort based on TCGA. *P*-values indicate the AUC of the combined *CDC20* and *PTGDS* model compared with that of the *CDC20* alone model or the *PTGDS* alone model.

To develop a more sensitive predictive tool, we assembled a two-gene panel combining *CDC20* and *PTGDS* based on the cohort from TCGA DLBC. Combination of *CDC20* and *PTGDS* had a better prognostic value compared with *CDC20* or *PTGDS* alone (AUC: 0.72 [95% confidence interval (CI): 0.61–0.79] compared with 0.62 [0.53–0.68], *P*=0.026; AUC: 0.72 [95% CI: 0.61–0.79] compared with 0.65 [0.57–0.72], *P*=0.039; Figure 7).

### *CDC20* expression is negatively regulated by DNA methylation

**Figure 8:**
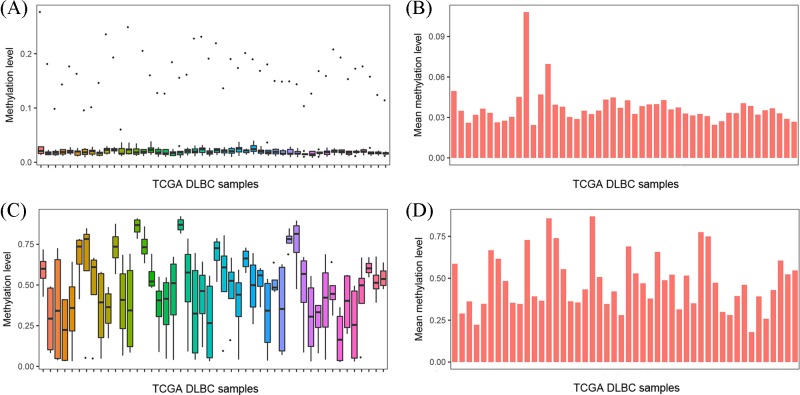
The methylation levels of CDC20 and PTGDS in the TCGA DLBC (**A**,**B**) For methylation array data, the β-value for CDC20 is illustrated as box plot and bar chart. (**C**,**D**) The box plot and bar chart for PTGDS methylation levels based on β-value(s).

**Figure 9:**
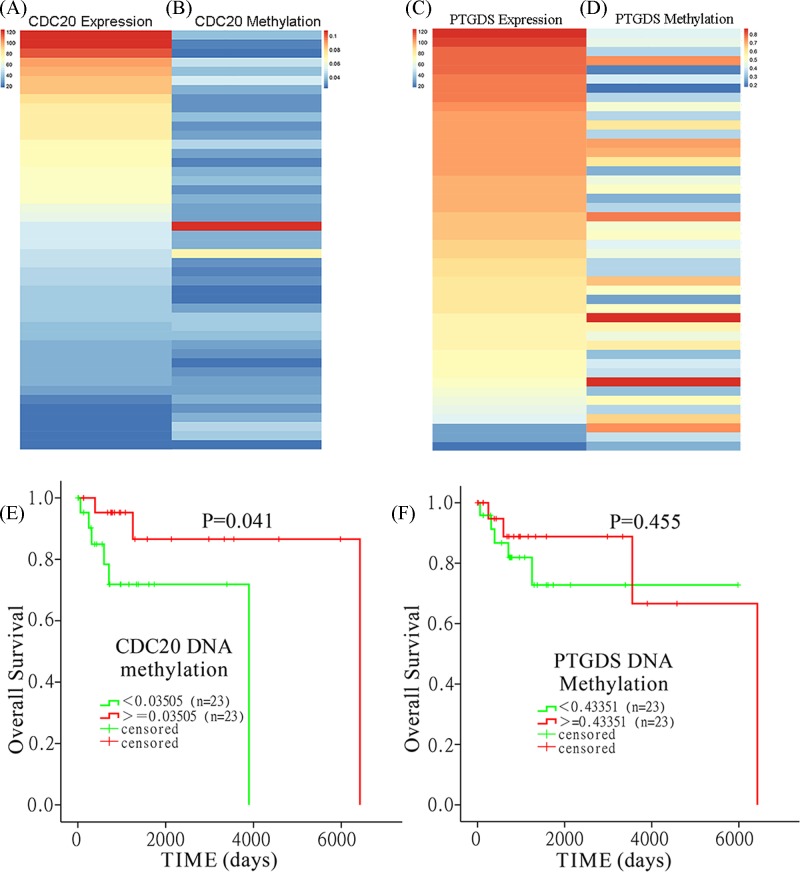
The DNA methylation levels of *CDC20* and *PTGDS* and their prognostic value in TCGA DLBC CDC20 expression is negatively regulated by DNA methylation: Heatmap of (**A**) *CDC20* expression and (**B**) *CDC20* DNA methylation from TCGA DLBC. Heatmap of (**C**) *PTGDS* expression and (**D**) *PTGDS* DNA methylation from TCGA DLBC. High *CDC20* methylation may be an indicator of favorable OS in patients with DLBCL. Associations between (**E**) CDC20 and (**F**) PTGDS DNA methylation levels and OS show that high *CDC20* methylation may be an indicator for favorable OS in TCGA DLBC.

We analyzed the 450 K methylation array data from TCGA DLBC to verify whether *CDC20* or *PTGDS* expression may be regulated by their DNA methylation status. By comparing *CDC20* and *PTGDS* DNA methylation levels, we found that the *CDC20* gene was hypomethylated in the DLBCL dataset (β-value: 0.0375 ± 0.0132, Figure 8A,B), while *PTGDS* was hypermethylated (β-value: 0.4744 ± 0.1676, Figure 8C,D). Furthermore, we confirmed that *CDC20* expression was reduced with increasing DNA methylation (Figure 9A,B), but *PTGDS* expression was not regulated by its DNA methylation status (Figure 9C,D).

### High *CDC20* methylation may be an indicator of favorable OS in patients with DLBCL

Based on the above results, high *CDC20* or low *PTGDS* expression may be a predictor for poor OS in DLBCL patients. We hypothesized that their methylation status may be associated with OS. Hence, we examined whether their methylation status was associated with OS. Compared with low *CDC20* methylation, patients with high *CDC20* methylation had significantly better OS (*P*=0.041) (Figure 9E). However, we failed to identity significant association between *PTGDS* methylation levels and OS (Figure 9F).

## Discussion

Abundant basic and clinical studies have tried to decipher the cause and underlying mechanisms for DLBCL. This has led to a serious challenge for the diagnosis and treatment of DLBCL. This may be due to the majority of the studies having focussed on a single molecular event [13] or that the results generated from single cohorts with genetic heterogeneity [14]. In this study, we integrated three DLBCL cohorts profile datasets covering different periods and countries. We utilized bioinformatics methods to comprehensively analyze these databases, and identified 153 overlapping DEGs with 74 up-regulated and 79 down-regulated genes. Furthermore, GO analysis indicated that the overlapped genes were mostly involved in cell cycle process, cell division, M phase, transcription, regulation of RNA metabolic process and purine nucleotide metabolic process at the BP level. Furthermore, the signaling pathway enrichment analysis revealed that the majority of the overlapped DEGs were enriched for cell cycle check points, APC-Cdc20-mediated degradation of Nek2A, cell cycle, EMT, oocyte meiosis, and hematopoietic cell lineage signaling pathway. Cell cycle, oocyte meiosis, and EMT were identified as the major pathways for the most significant module of the overlapped DEGs. Our study offers new insights into the molecular mechanisms of tumorigenesis and progression of DLBCL and identified hub genes that may be potential therapeutic or diagnostic targets for DLBCL.

In recent years, integrated bioinformatics analysis has been progressively used for understanding cancer pathogenesis, development of potential biomarkers and molecular target therapies for diagnosis, and for the prognostication and treatment for various cancers. Ma et al. [15] identified *CXCR4* as a potential biomarker for glioblastoma multiforme using integrated bioinformatics analysis and found that low expression of *CXCR4* may indicate favorable OS for GBM patients. In addition, another study showed that the expression of *BUB1B* and *CENPF* were up-regulated in nasopharyngeal carcinoma and their high expression was associated with poor OS [16]. Similar studies have also been reported for DLBCL. Song et al. demonstrated that *CD59* could predict response and outcome of DLBCL patients treated with R-CHOP in a single cohort study [14]. Another study identified a single molecular biomarker for the diagnosis and treatment of DLBCL using bioinformatics analysis [13]. However, compared with our study, their studies only analyzed gene expression profiles or only identified a single biomarker to predict survival. In our study, hub genes were selected by the degree of connectivity. We integrated three gene expression profiles, and then combined the results of MCODE and PPI to identify hub genes. Furthermore, we developed a *CDC20–PTGDS* combination panel to predict OS with more sensitively. Finally, our model was validated using TCGA DLBC and another independent cohort GSE31312 to increase confidence of our model.

In our study, six hub genes, i.e. *CDC20, MELK, PBK, PTGDS, PCNA*, and *CDK1* were narrowed down, of which, *CDC20* and *PTGDS* were the most significantly associated with OS. A number of these genes have been reported as biomarkers in previous studies. *CDC20* is an oncogene that plays a pivotal role in mitotic progression. Suppressing the activity of *CDC20* regulates the cell cycle and promotes apoptosis [17]. Wu et al. [18] reported that *CDC20* was highly expressed in colorectal cancer and concluded that *CDC20* was a predictor for adverse clinical outcomes and an independent prognostic factor. Kidokoro et al. [19] demonstrated that *CDC20* repression mediates the tumor suppressive function of *p53*. These findings are in-line with *p53* inactivation observed in various cancer tissues including glioma, lung cancer, and breast ductal carcinoma and is likely due to *CDC20* up-regulation [19]. In human adult T-cell-leukemia (ATL) cells, studies have demonstrated that APC^CDC20^ is a physiological E3 ligase that promotes the ubiquitination and destruction of the tumor suppressor, Bim, thus conferring resistance of cancer cells to chemoradiation.

Our study provides a rationale for developing specific *CDC20* inhibitors as efficient anticancer agents [20]. Although several studies have assessed the role of *CDC20* for the initiation and progression of several human cancers including colorectal cancer, lung cancer, glioma, and ATL, few have studied the role of *CDC20* in DLBCL. We verified the expression of *CDC20* in DLBCL tissue was significantly higher compared with normal tissue, and patients with high *CDC20* levels had an adverse prognosis. The expression level and clinical function of *CDC20* in DLBCL is consistent with previous studies.

Several recent studies have demonstrated that *PTGDS* has important vascular functions [21] as well as being associated with cancer [22,23]. Several other studies have reported that *PTGDS* expression is lower in gastric cancer tissues compared with *PTGDS*, which was associated with better prognosis [24]. Similar results have also been observed in lung cancer [25]. Our study is the first to demonstrate the expression pattern of *PTGDS* and its prognostic value for DLBCL patients. In-line with previous studies, the expression level of *PTGDS* in our study was significantly lower in DLBCL compared with normal tissue. Based on survival analysis, we also demonstrated that low *PTGDS* expression levels were significantly correlated with poor OS in DLBCL patients based on data from TCGA DLBC and an independent cohort GSE31312 dataset.

EMT has been shown to enhance solid tumor metastasis, invasion, and proliferation [26]. Omori et al. [27] reported that endothelial *PTGDS* deficiency could lead to accelerated vascular hyperpermeability, angiogenesis, and EMT in tumors, which in turn reduced tumor cell apoptosis. In our study, pathway enrichment analysis demonstrated that *PTGDS* was enriched in EMT and was in-line with the above-mentioned studies in which deficiency in *PTGDS* induced EMT. Lemma et al. [26] showed that EMT is also present in lymphomas. Both ZEB1 and Slug, EMT-mediating transcription factors (TFs), were highly expressed and associated with adverse prognosis in DLBCL. Taken together, we hypothesized that *PTGDS* inhibits EMT and suppresses tumor proliferation and invasion by regulating the expression of *ZEB1* and *Slug* in DLBCL. Future experiments need to be performed to verify this hypothesis.

Recent studies suggest that DNA repair enzyme MGMT methylation is a significant prognostic factor for patients with DLBCL [28,29]. However, the prognostic role of *CDC20* and *PTGDS* methylation levels in DLBCL has never been reported previously. By data mining using the DLBCL cohort in the TCGA database, *CDC20* hypomethylation and *PTGDS* hypermethylation were observed in DLBCL patients. We demonstrated that *CDC20* expression was negatively regulated by its DNA methylation, whereas *PTGDS* was not affected by its methylation status. DNA promoter methylation may have prognostic value for DLBCL [30]. We examined the prognostic value of *CDC20* promoter methylation in DLBCL and found that favorable OS was observed in patients with high *CDC20* promoter methylation (*P*=0.041). This suggests that *CDC20* promoter methylation status may be a biomarker for predicting OS in patients with DLBCL.

Combined predictive models for OS is superior to predictive models relying on a single predictor [31]. Liu et al. [32] reported that combining two independent prognostic factors, i.e. a five miRNA signature and TNM stage, was a more sensitive predictor for nasopharyngeal carcinoma. In another study, the *FGD3-SUSD3* metagene model was demonstrated to have a superior prognostic value for breast cancer [33]. Our two genes combined panel for DLBCL, in which low *CDC20* expression and high *PTGDS* expression, had a superior prognostic value compared with *CDC20* or *PTGDS* alone. This *CDC20-PTGDS* combined model could allow clinicians to identify high-risk patients and lead to a more personalized treatment strategy for patients with DLBCL.

## Conclusion

In summary, *CDC20* and *PTGDS* were identified from the DEGs and *CDC20* overexpression and *PTGDS* low expression were associated with poor prognosis. *CDC20* expression is negatively regulated by DNA methylation in DLBCL and its hypomethylation may be a potential indicator for adverse OS. In addition, our study demonstrated that cell cycle, oocyte meiosis, and EMT were potential mechanisms and pathways associated with DLBCL. However, we need to perform additional experiments to verify our results generated from our bioinformatics analysis.

It has been reported that a single biomarker or pathway is not sufficient to explain cancer pathogenesis because of the complex molecular mechanisms that govern oncogenesis [34]. Hence, we assembled a prognostic score model combining the expression levels of *CDC20* and *PTGDS*. This combined gene expression model was more sensitive with higher predictive power. In our present study, prognostic signatures including *CDC20* and *PTGDS* were identified from the DEGs and could predict OS in DLBCL patients, which will provide useful guidance for therapeutic applications.

## Data availability

All data were extracted from previously published freely available datasets.

## Abbreviations

APC: anaphase promoting complex
ATL: adult T-cell-leukemia
AUC: area under ROC curve
BP: biological process
CC: cellular component
Cdc20: cell division cycle 20
CI: confidence interval
DAVID: Database for Annotation, Visualization and Integrated Discovery
DEG: differentially expressed gene
DLBCL: diffuse large B-cell lymphoma
EMT: epithelial-to-mesenchymal transition
GBM: glioblastoma multiforme
GEO: Gene Expression Omnibus
GO: gene ontology
LogFC: log fold change
MCODE: Molecular Complex Detection
MF: molecular function
NGS: next-generation sequencing
OS: overall survival
PPI: protein–protein interaction
PTGDS: prostaglandin D2 synthase
ROC: receiver operating characteristic
STRING: Search Tool for the Retrieval of Interacting Genes
TCGA: The Cancer Genome Atlas
